# Free-Space QKD with Modulating Retroreflectors Based on the B92 Protocol

**DOI:** 10.3390/e24020204

**Published:** 2022-01-28

**Authors:** Minghao Zhu, Min Hu, Banghong Guo

**Affiliations:** 1 Guangdong Provincial Key Laboratory of Nanophotonic Functional Materials and Devices, Guangdong Provincial Key Laboratory of Quantum Engineering and Quantum Materials, South China Normal University, Guangzhou 510006, China; 15888900144@163.com; 2 National Quantum Communication (Guangdong) Co., Ltd., Zhaoqing 526238, China; 3 Key Laboratory of Quantum Information, University of Science and Technology of China, Chinese Academy of Sciences, Hefei 230026, China

**Keywords:** quantum key distribution, free-space, modulating retroreflector, B92 protocol

## Abstract

Free-space quantum key distribution (QKD) has attracted considerable attention due to its lower channel loss and link flexibility. It allows two participants share theoretical unconditional secure keys, and can potentially be applied to air-to-ground quantum communication to establish a global quantum network. Free-space QKD using modulating retro-reflectors (MRR-QKD) significantly reduces the pointing requirement and simplifies the structure of the mobile terminal, therefore making it suitable for lightweight aircraft such as unmanned aerial vehicle and Cubesat, etc. Based on intensity modulation of two non-orthogonal states and the B92 protocol, we proposed a scheme to improve the previous work (*Optics Express* **2018**, *26*, 11331). Our scheme simplifies the optical structure and shows more robustness in equipment imperfection. The analysis and simulation show that the number of multiple quantum well modulators needed in our scheme decreases from eight to three with similar performance. Additionally, while the previous scheme cannot work due to low modulator extinction ratio or high optical misalignment, our scheme can still operate.

## 1. Introduction

Quantum key distribution (QKD) can help two distant parties to realize theoretical unconditional security communication [1]. QKD can be categorized into fiber-based QKD and free-space QKD according to different communication channels. The longest transmission distance that fiber-based QKD can achieve is only hundreds of kilometers because of the intrinsic loss of the fiber (approximately 0.2 dB/km@1550 nm) [2,3,4]. Conversely, free-space QKD can achieve a transmission distance of thousands of kilometers owing to its lower channel loss, and it is also suitable for mobile communication owing to its flexible link [5,6,7]. A worldwide quantum-secured communication network is foreseeable by integrating free-space and fiber-based QKD [8].

Despite encouraging advances in free-space QKD [9,10], many challenges remain, for example, the contradiction between the limited payload of satellites and the complex structure of free-space QKD system [6]. The miniaturization and light weight of QKD systems has become an important topic [11,12]. Cheaper microsatellites, such as standardized CubeSats, will play a greater role in future quantum networks [13,14,15]. Free-space QKD using modulating retro-reflectors (MRR-QKD) significantly reduces the pointing requirement and simplifies the structure of the mobile terminal, therefore decreasing the payload greatly, which may be a prospective scheme.

In the MRR-QKD system, two communication parties are defined as the interrogator and MRR terminal. Compared with the traditional QKD system, the structure of the MRR terminal in the MRR-QKD system is simpler, and the pointing requirement is reduced significantly(more than three orders of magnitude) [16]. The concept of MRR-QKD was first proposed by Rarity in 2002 [17]. Vallone demonstrated an imperfect experiment for satellite-to-ground MRR-QKD between low earth orbit (LEO) satellites and the Matera Laser Ranging Observatory (MLRO) [18]. Many studies have been conducted recently [16,19,20]. In 2017, Rabinovich designed a practical MRR-QKD system and achieved a quantum bit error rate (QBER) of approximately 5% with a modulation rate of 1 MHz in the laboratory [16].

The MRR-QKD system of Rabinovich [16], which we will call RM2018 in this paper, uses up to eight multiple quantum wells (MQW) [21,22] intensity modulators to realize polarization modulation. Owing to the finite extinction ratio [23] of MQW modulators, there is leakage noise in this system, which causes higher QBER. Based on the superposition of two mutually perpendicular waves [24], we proposed a MRR-QKD system based on the B92 protocol [25]. Our scheme reduced the complexity of the system structure and eliminated leakage noise. The remainder of this paper is organized as follows. In Section 2, we introduce the configuration of our MRR-QKD system and its mathematical model. In Section 3, the performance of the system under different extinction ratios and optical misalignment errors is studied and compared with previous works. In Section 4, we discuss our results. In Section 5, we present our conclusions.

## 2. MRR-QKD with B92

First, let us review the scheme proposed in RM2018. Four pairs of MQWs were used to realize polarization modulation, where each pair corresponded to one of the four BB84 states (0, 45, 90, and $135^{{}^{\circ}}$). While the transmission loss of the MQW modulator is set to the lowest (highest), the MQW is in the “on” (off) state. The polarization modulation is realized by controlling the states of MQWs. For example, when the MRR terminal sends a horizontal state, the pair of MQWs corresponding to the horizontal state will be “on” and the others will be “off”. More details are shown in Appendix C.

The extinction ratio of the MQW bewteen the “on” and “off” states is only approximately 7.5 dB, which is too small. Unwanted light will leak from the MQW, which are “off”, resulting in extra QBER. To decrease the QBER, RM2018 uses a stack of two MQWs packaged together to achieve an extinction ratio *r* of 15 dB in every path. However, this will greatly increase the complexity and cost of the system, and the extinction ratio will remain unsatisfactory.

**Figure 1:**
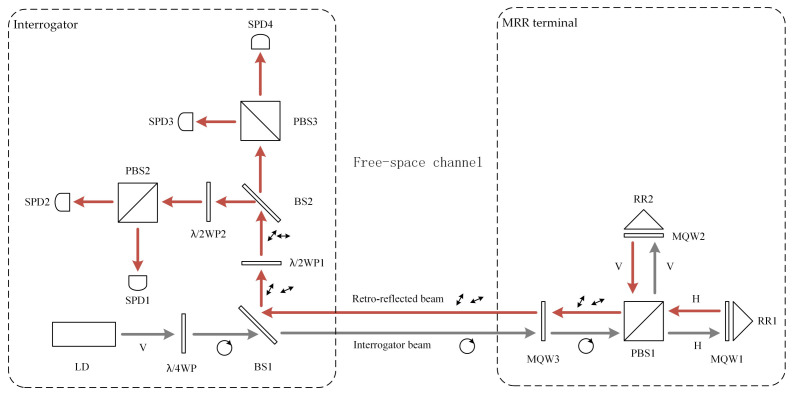
Schematic of the modulating retroreflector-quantum key distribution (MRR-QKD) system. LD: Laser Diode; WP: Wave Plate; BS: Beam Splitter; PBS: Polarizing Beam Splitter; SPD: Single Photon Detector; RR: Retro-reflector.

Our MRR-QKD system, based on the superposition principle of two mutually perpendicular waves, uses only three MQW modulators in total and eliminates unexpected noise leakage. The configuration of the MRR-QKD system is shown in Figure 1.

The system works as follows. First, the interrogator generates an interrogation beam, which is a classical light pulse with right-hand circular polarization the same with RM2018, and sends it to the MRR terminal through a free-space channel. After arriving at the MRR terminal, the interrogation beam passes through MQW3, which is used to modulate decoy states. Then, it is split into two beams with the same intensity by a PBS, the horizontal state is transmitted, and the vertical state is reflected. These two beams will pass forward through an MQW and be reflected by a retroreflector, and then pass backward through the MQW again. Therefore, the intensity of these two beams are modulated by MQW1 (and MQW2) twice. Finally, they are combined by the PBS, and the output is a linearly polarized beam with polarization direction θ (see Appendix A).

(1) $$|\psi_{out}\rangle =|\beta_{\theta }\rangle =|\cos\theta \beta_{H}\rangle |\sin\theta \beta_{V}\rangle$$

where $|\beta_{\theta }\rangle$ is the linearly polarized coherent state. β is the amplitude of the beam, and $\theta =\arctan(\sqrt{M_{2}/M_{1}})$ is its polarization direction. $M_{1}$ and $M_{2}$ are the modulation efficiencies of MQW1 and MQW2, respectively.

The B92 protocol is adopted in our system. Each retroreflected beam is in one of two linear polarizations with polarization directions of $22.5^{{}^{\circ}}$ and $67.5^{{}^{\circ}}$ by adjusting the ratio of the modulation efficiency of MQW1 and MQW2. This is slightly different from the general B92 protocol.

The beam is attenuated and modulated by MQW3 to generate signal or decoy states. After the beam is transmitted back and received by the interrogator, it is still linearly polarized because of the stability of the polarization in free space [26]. However, its polarization direction is changed because of the relative orientation of the interrogator and the MRR terminal. A half-wave plate is used for polarization compensation. The incident beam will be converted to $0^{{}^{\circ}}$ ($45^{{}^{\circ}}$) linear polarization. The following procedures, such as measurement and post-processing, are the same as those of the general B92 protocol [25,27].

## 3. Results

We now analyze the performance of our MRR-QKD system proposed in Section 2 and compare it with RM2018. With two decoy state technology, the secure key rate (SKR) of the QKD system is [28].

(2) $$R=q\{-fQ_{\mu }H_{2}\left(E_{\mu }\right)+Q_{1}^{L,\nu_{1},\nu_{2}}\left[1-H_{2}\left(e_{1}^{U,\nu_{1},\nu_{2}}\right)\right]\}\;$$

where *q* is the protocol efficiency, *f* is the bi-directional error correction efficiency, $Q_{\mu }$ is the gain of the signal state, $E_{\mu }$ is the overall QBER, $Q_{1}^{L,\nu_{1},\nu_{2}}$ and $e_{1}^{U,\nu_{1},\nu_{2}}$ are the lower bound of the gain and upper bound of the error rate for single photon state, respectively. $H_{2}\left(\cdot \right)$ is the binary Shannon information function.

In our system, the protocol efficiency of the B92 protocol is q=1/4, whereas BB84 is used in RM2018, q=1/2. The intensity of the signal state is μ. The maximum intensity range of the decoy state is ν∈[μ/r,μ). In the asymptotic case, the smaller the value of $\nu_{1}+\nu_{2}$, the higher the SKR [28]. Therefore, we chosen the signal state intensity μ=0.48, decoy state intensity $\nu_{1}=\mu /r$ and vacuum state $\nu_{2}=0$. Other simulation parameters are listed in Table 1.

**Figure 2:**
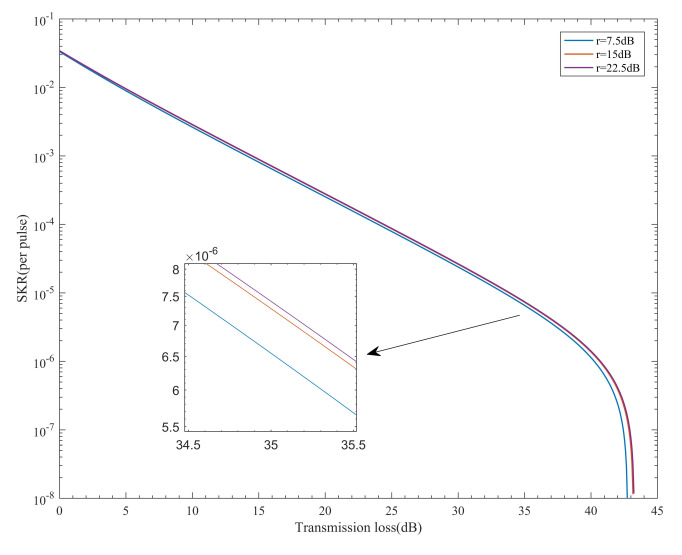
The relationship between the Secure key rate(SKR) and the transmission loss under different extinction ratios.

In Section 2, we showed that the extinction ratio of the MQW would cause leakage noise while the MQW is “off” in RM2018. This leakage noise would make the QBER increase. The smaller the extinction ratio, the higher the leakage noise. In our scheme, the extinction ratio does not generate leakage noise but only restricts the intensity of the decoy states. We demonstrated the extinction ratio effect in our system, the simulation results are shown in Figure 2.

The three curves with extinction ratios of 7.5, 15, and 22.5 dB overlap almost completely. This indicates that the extinction ratio does not have a significant impact on the SKR of our scheme. This is because the SKR is not sensitive to the intensity of the decoy state. Additionally, in contrast to RM2018, which stacks two MQWs to increase the extinction ratio, our system requires only one MQW in each path.

**Figure 3:**
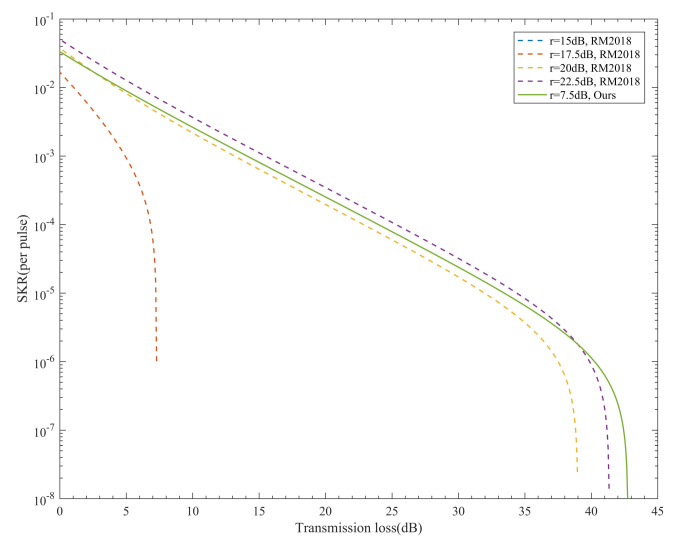
The relationship between the SKR and the transmission loss of our system and RM2018 under different extinction ratios. The dotted line represents the RM2018, and the solid line represents our system. The blue dotted line vanishes because the SKR is lower than zero.

Under different extinction ratios, we compare the SKR of our system and the RM2018. The results are shown in Figure 3. Considering the additional QBER resulting from noise leakage for the finite extinction ratio, the probability of a photon clicks the erroneous detector should be corrected to $e_{detect}^{\prime }=\frac{r-1}{r+3}e_{detect}+\frac{2}{r+3}$ [23]. $e_{detect}$ is the probability that a photon clicks the erroneous detector due to the optical misalignment and *r* indicates the extinction ratio.

As shown in Figure 3, the SKR of RM2018 is very sensitive to the extinction ratio. The SKR decreases as the extinction ratio reduces. When the extinction ratio was less than 15 dB, the SKR of RM2018 decreased below zero (vanishing blue dotted line). The performance of our system with only a 7.5 dB extinction ratio could reach at least 70% of RM2018 with a 22.5 dB extinction ratio, and even higher for the maximum transmission distance. This means that only one MQW is needed for each path in our system, whereas RM2018 requires three MQWs.

**Figure 4:**
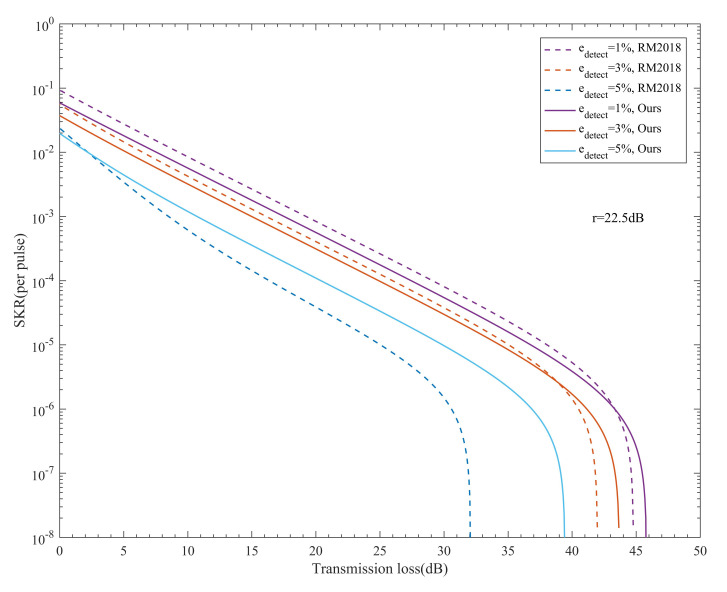
The relationship between the SKR and the transmission loss of our system and RM2018 under different $e_{detect}$. The dotted line represents RM2018, and the solid line represents our system.

In RM2018, $e_{detect}^{\prime }$ is related not only to the extinction ratio, but also the optical misalignment error $e_{detect}$. As shown in Figure 4, we studied the relationship between the SKR and transmission loss of both our system and RM2018 under different $e_{detect}$ values. The dotted line represents RM2018, and the solid line represents our system.

When the extinction ratio is fixed, it is clear that the SKR of the two systems decreases as $e_{detect}$ increases. We found that the robustness of our scheme countering the increase in $e_{detect}$ was better than that of RM2018. When $e_{detect}=1\%$ and 3%, the SKR of RM2018 was slightly higher than that of our system in most cases, mainly because the efficiency of the BB84 protocol was twice that of the B92 protocol. However, when $e_{detect}=5\%$, the performance of our system was much greater than that of RM2018. This result shows that the higher the $e_{detect}$, the greater the advantage of our system is over the RM2018. This occurs because the SKR decreases faster as $e_{detect}$ increases. Moreover, the $e_{detect}^{\prime }$ of RM2018 is always larger than the $e_{detect}$ of our system.

**Figure 5:**
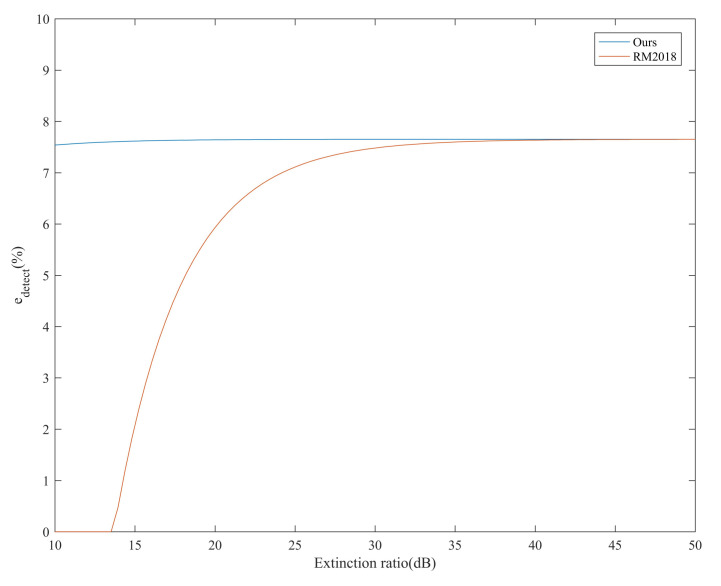
The relationship between the extinction ratio and the maximal tolerance of misalignment-error probability $e_{detect}^{U}$ in transmission probability of 100%.

We study the intrinsic maximal tolerance of misalignment error $e_{detect}^{U}$ at which the SKR becomes zero. The relationship between the extinction ratio and $e_{detect}^{U}$ of two systems is compared in the condition of transmission probability of 100%. The result is shown in Figure 5. We can see that $e_{detect}^{U}$ in our system is not sensitive to the change of the extinction ratio. The change of it is mainly originated in the change of decoy state intensity. When the extinction ratio is lower than about 13.5 dB, RM2018 cannot operate. This is because the error rate caused by noise leakage has exceeded the threshold. In other cases, $e_{detect}^{U}$ climbs up with the increase of the extinction ratio and the difference between our system and RM2018 gets smaller. When the extinction ratio is greater than 40 dB, they almost have similar performance $e_{detect}^{U}\approx 7.65\%$.

## 4. Discussion

In the previous sections, we analyzed our MRR-QKD scheme with the B92 protocol and compared it with RM2018. The results are listed in Table 2. Our scheme requires only three MQW modulators, which is significantly less than the eight in RM2018. Two of them are used for polarization modulation and the other is used for decoy state generation. Based on the superposition principle of perpendicular polarization states, we make full use of each beam to solve the problem of extra QBER on account of the finite extinction ratio. This could effectively improve the performance of the MRR-QKD system. Although the protocol efficiency was only half that of RM2018, our scheme could reach a much higher SKR, while having a high optical misalignment error or low extinction ratio.

These two systems both modulate the polarization of the beam by means of intensity modulation. The modulation rate of the MQW intensity modulator can be up to 100 MHz [16]. The low extinction ratio of the MQW will cause higher QBER in RM2018. The protocol efficiency in our system is half that in RM2018 because of the principle of the modulation method. In the ideal circumstance of the infinite extinction ratio, the performance of RM2018 is better than ours. But if the MQW intensity modulator for polarization modulation are replaced by phase modulators, it is possible to apply the BB84 protocol in our system [30]. Thus, we can achieve the same performance as RM2018 with smaller cost. The decoding system of the B92 protocol in our system is the same as that of RM2018 and very close to the classical free-space QKD system based on the BB84 protocol [6,14,31]. This means that it is convenient to fit our system into the current free-space QKD network [31,32].

The imperfection of the device, such as misalignments and detection efficiency mismatch, will allow eavesdroppers to attack in side channels. These flaws have been proved not affecting the security of the system within several counter-measures(temporal, spatial and frequency filtering and so on) [33]. The signal in the MRR-QKD system makes a round-trip through atmospheric channels. The transmission process is similar to the “plug-play” system in fiber channels [34]. The source of such two-way QKD systems is untrusted; its security has been proven [35,36]. Unlike the “plug-play” system, the MRR-QKD system based on free-space channels will be subject to the off-axis attack. This attack can be resisted by monitoring the incident angle of the interrogation beam and using modulators with pixel units to code beams with authentication. Therefore, those modulators incapable of pixelated modulation are not suitable to be used in MRR-QKD system, like AOMs modulators with comparable modulation rate and higher extinction ratio [37]. Other attacks, like photon number splitting, intercept-resend and on-axis attacks, are also explicated [16].

## 5. Conclusions

We propose an MRR-QKD scheme using the B92 protocol. Based on the superposition principle of perpendicular polarization states, this system realizes polarization modulation by means of intensity modulation. Compared with RM2018, our system not only simplifies the structure, but also improves the tolerance of the MRR-QKD system for extinction ratio and optical misalignment error. This novel, cheaper and smaller system can be expected to be widely applied in mobile platforms with limited payloads, such as cube satellites, unmanned aerial vehicles, automobiles, etc.

## Figures and Tables

**Table 1:** List of Simulation Parameters [29].

Symbol	Description	Value
λ	wavelength	1550 nm
$e_{detect}$	misalignment-error probability ^1^	3.3%
$Y_{0}$	background rate	$1.7\times 10^{-6}$
$\eta_{d}$	detection efficiency ^2^	1
*f*	error correction efficiency	1.22

^1^ Due to imperfect polarization compensation, assembling misalignment, etc.; ^2^ The detection efficiency is considered in transmission loss.

**Table 2:** Comparison of our scheme and RM2018 [16].

Items	RM2018	Our Scheme	Condition
Complexity	high	low	
Multiple quantum wells	8	3	
Protocol	BB84	B92	
SKR ^1^	$3.21\times 10^{-5}$	$2.65\times 10^{-5}$	r=22.5 dB
	$1.71\times 10^{-5}$	$2.65\times 10^{-5}$	r=20 dB
	0	$2.64\times 10^{-5}$	r=17.5 dB
	0	$2.61\times 10^{-5}$	r=15 dB
SKR ^2^	$8.05\times 10^{-5}$	$5.44\times 10^{-5}$	$e_{detect}=1\%$
	$3.79\times 10^{-5}$	$3.00\times 10^{-5}$	$e_{detect}=3\%$
	$1.50\times 10^{-6}$	$9.69\times 10^{-6}$	$e_{detect}=5\%$

^1^ Transmission loss is 30 dB, error probability of optical misalignment is 3.3%. ^2^ Transmission loss is 30 dB, extinction ratio of each path is 22.5 dB.

**Figure A1:**
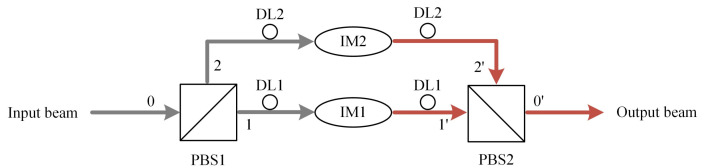
Equivalent schematic of the MRR terminal with unidirectional transmission.

**Figure A2:**
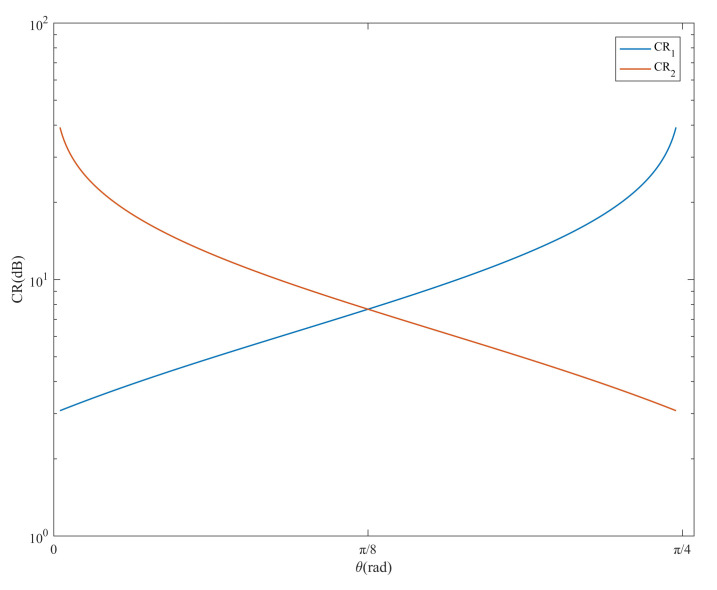
The contrast ratios of two paths while modulating different polarizations.

**Figure A3:**
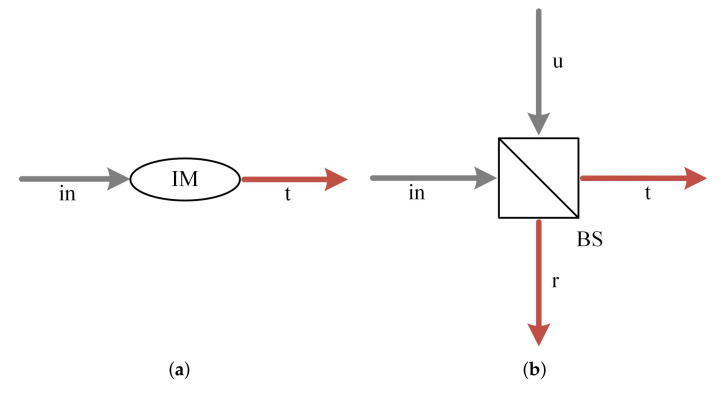
Schematic of beams passing through: (**a**) intensity modulator; (**b**) beam splitter.

**Figure A4:**
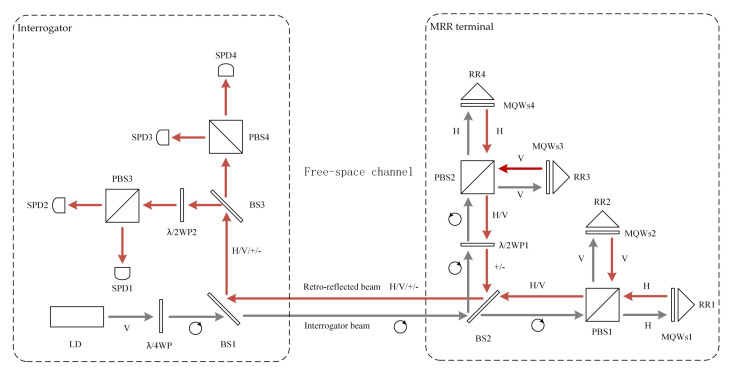
Schematic of the MRR-QKD system in RM2018 [16].

## Data Availability

Not applicable.

## Appendix A

For ease of analysis, we transformed the round-trip MRR terminal in Figure 1 into a unidirectional transmission model, as shown in Figure A1. MQW3 was omitted because it does not affect the result.

The interrogation beam incident on the MRR terminal is a coherent state with right-handed circular polarization.

(A1) $$|\psi_{0}\rangle ={|\alpha_{R}\rangle }_{0}$$

where α is the amplitude of the coherent state, and the subscript 0 indicates the transmission path shown in Figure A1.

Any pure coherent state can be expressed as the direct product state of two coherent states, whose polarizations are orthogonal to each other.

(A2) $$|\psi_{0}\rangle =D\left(\alpha_{R}\right)|0\rangle ={|\frac{1}{\sqrt{2}}\alpha_{H}\rangle }_{0}{|\frac{1}{\sqrt{2}}e^{i\frac{\pi }{2}}\alpha_{V}\rangle }_{0}$$

The right-handed circular polarization coherent state can be expressed as the direct product of the horizontal and vertical polarization coherent states. Their intensity are equal, which is half of the interrogation beam, and the phase difference is π/2.

Because the PBS transmits only the horizontal $|H\rangle$ and reflects the vertical $|V\rangle$ polarizations, after passing through PBS1, the interrogation beam will evolve into

(A3) $$|\psi_{1}\rangle ={|\frac{1}{\sqrt{2}}\alpha_{H}\rangle }_{1}{|\frac{1}{\sqrt{2}}e^{i\frac{\pi }{2}}\alpha_{V}\rangle }_{2}$$

The phase shift generated by PBS has been ignored because it does not influence the conclusion. The beam transmitted along Paths 1 and 2 will pass through a free-space path, which is equivalent to a phase shifter. Then the joint state before incident $IM_{1}$ and $IM_{2}$ is:

(A4) $$\begin{array}{cc} |\psi_{2}\rangle & ={\hat{P}}_{1}{\hat{P}}_{2}|\psi_{1}\rangle \\ \; & =e^{i\theta_{1}{\hat{n}}_{1}}e^{i\theta_{2}{\hat{n}}_{2}}{|\frac{1}{\sqrt{2}}\alpha_{H}\rangle }_{1}{|\frac{1}{\sqrt{2}}e^{i\frac{\pi }{2}}\alpha_{V}\rangle }_{2}\\ \; & ={|\frac{1}{\sqrt{2}}e^{i\theta_{1}}\alpha_{H}\rangle }_{1}{|\frac{1}{\sqrt{2}}e^{i\left(\frac{\pi }{2}+\theta_{2}\right)}\alpha_{V}\rangle }_{2} \end{array}$$

where ${\hat{P}}_{1}$ is the phase shifter operator acting on Path 1, ${\hat{n}}_{1}$ is the photon number operator acting on Path 1.

Afterward, these two beams are modulated by intensity modulator $IM_{1}$ and $IM_{2}$ respectively. The output state is as follows (for details, see Appendix B).

(A5) $$|\psi_{3}\rangle ={|\frac{1}{\sqrt{2}}\sqrt{M_{1}}e^{i\theta_{1}}\alpha_{H}\rangle }_{1^{\prime }}{|\frac{1}{\sqrt{2}}\sqrt{M_{2}}e^{i\left(\frac{\pi }{2}+\theta_{2}\right)}\alpha_{V}\rangle }_{2^{\prime }}$$

Then the beam passes through the free-space path again, and we have

(A6) $$|\psi_{4}\rangle ={|\frac{1}{\sqrt{2}}\sqrt{M_{1}}e^{i2\theta_{1}}\alpha_{H}\rangle }_{1^{\prime }}{|\frac{1}{\sqrt{2}}\sqrt{M_{2}}e^{i\left(\frac{\pi }{2}+2\theta_{2}\right)}\alpha_{V}\rangle }_{2^{\prime }}$$

Finally, these two beams are combined by the PBS

(A7) $$|\psi_{out}\rangle ={|\frac{1}{\sqrt{2}}\sqrt{M_{1}}e^{i2\theta_{1}}\alpha_{H}\rangle }_{0^{\prime }}{|\frac{1}{\sqrt{2}}\sqrt{M_{2}}e^{i\left(\frac{\pi }{2}+2\theta_{2}\right)}\alpha_{V}\rangle }_{0^{\prime }}$$

If $2\theta_{1}=\pi /2+2\theta_{2}$, $|\psi_{out}\rangle$ is converted to

(A8) $$|\psi_{out}\rangle =|\frac{1}{\sqrt{2}}\sqrt{M_{1}}\alpha_{H}\rangle |\frac{1}{\sqrt{2}}\sqrt{M_{2}}\alpha_{V}\rangle$$

It is the direct product state of the horizontal and vertical polarization coherent states, which is linearly polarized. If $\sqrt{M_{1}}=C\cos\theta$, and $\sqrt{M_{2}}=C\sin\theta$, the state is

(A9) $$|\psi_{out}\rangle =|C^{\prime }cos\theta \alpha_{H}\rangle |C^{\prime }sin\theta \alpha_{V}\rangle =|cos\theta \beta_{H}\rangle |sin\theta \beta_{V}\rangle =|\beta_{\theta }\rangle$$

where $\beta =C^{\prime }\alpha$ is the amplitude and θ is the polarization direction of the output beam.

The polarization of the beam depends on the intensity of two combined beams, satisfying $\theta =\arctan(\sqrt{M_{2}/M_{1}})$. We can adjust the ratio of $M_{1}$ and $M_{2}$ to realize different linear polarizations. However, only a few polarizations can be achieved in this manner. While the phase difference is zero, only linear polarizations with axes θ∈(0,π/2) are possible. Therefore, we use the B92 protocol, which requires only two states with polarization direction θ and $\theta +45^{{}^{\circ}}$ instead of the BB84 protocol.

When the polarization direction of the output beam is θ, the corresponding modulation efficiency of MQW1 and MQW2 are $\sqrt{M_{1}}=C\cos\theta$ and $\sqrt{M_{2}}=C\sin\theta$, respectively. While the polarization direction is $\theta +45^{{}^{\circ}}$, $\sqrt{M_{1}}=C\cos\left(\theta +45^{{}^{\circ}}\right)$ and $\sqrt{M_{2}}=C\sin\left(\theta +45^{{}^{\circ}}\right)$. The contrast ratio (CR) of MQW1 and MQW2 while modulating the different polarizations are

(A10) $$CR_{1}=\frac{M_{1}}{M_{1}^{\prime }}=\frac{\cos^{2}\theta }{\cos^{2}\left(\theta +45^{{}^{\circ}}\right)},\;CR_{2}=\frac{M_{2}^{\prime }}{M_{2}}=\frac{\sin^{2}\left(\theta +45^{{}^{\circ}}\right)}{\sin^{2}\theta }$$

As shown in Figure A2, $CR_{1}$ and $CR_{2}$ exhibit opposite trends while θ increases. When θ=π/8, $CR_{1}=CR_{2}\approx 7.6$ dB, which is approximately equal to the extinction ratio of a MQW [18]. These correspond to $22.5^{{}^{\circ}}$ and $67.5^{{}^{\circ}}$ linear polarization, respectively. Therefore, we used these two polarizations.

According to the superposition principle of two perpendicular polarization states, we can see that the polarization state of the retroreflected beam is dependent on the intensity and phase difference of two beams inputting the PBS. We modulated the intensity of the beam by adjusting the modulation efficiency of the MQW to obtain the expected polarization while keeping the phase difference constant over a fixed optical path. Only one MQW modulator in every path and two MQW modulators in total for polarization modulation were required. However, it is worth noting that only a few polarizations can be achieved in this manner. Therefore, the B92 protocol which uses two nonorthogonal states was adopted in our system. Additionally, MQW3 was used for the decoy state, and there were only three MQW modulators in our system, which is less than the eight required by RM2018.

## Appendix B

The signal passing through the intensity modulator is equivalent to the signal passing through the beam splitter. The transmittance *t* (or reflectivity *r*) of the beam splitter is equal to the modulation efficiency *M* of the intensity modulator, as shown in Figure A3.

The beam splitter can be expressed as follows:

(A11) $$\left\{\begin{array}{c} a_{in}^{+}=\sqrt{r}a_{r}^{+}+\sqrt{1-r}a_{t}^{+}\\ a_{u}^{+}=\sqrt{1-r}a_{r}^{+}-\sqrt{r}a_{t}^{+} \end{array}\right.$$

The coherent state incidents the “in” port of the intensity modulator and exists from the “*t*” port. This model is equivalent to the vacuum state incidents the “*u*” port of the beam splitter, the coherent state incidents the “in” port and the combined beam exists from the “*t*” port.

(A12) $$\begin{array}{c} {|\alpha \rangle }_{in}{|0\rangle }_{u}=D\left(\alpha \right){|0\rangle }_{in}{|0\rangle }_{u}=\exp\left(\alpha a_{in}^{+}-\alpha^{*}a_{in}\right){|0\rangle }_{in}{|0\rangle }_{u}\\ \to \exp\left(\alpha \left(\sqrt{r}a_{r}^{+}+\sqrt{1-r}a_{t}^{+}\right)-\alpha^{*}\left(\sqrt{r}a_{r}+\sqrt{1-r}a_{t}\right)\right){|0\rangle }_{r}{|0\rangle }_{t}\\ =\exp\left(\sqrt{r}\alpha a_{r}^{+}-\sqrt{r}\alpha^{*}a_{r}\right)\exp\left(\sqrt{1-r}\alpha a_{t}^{+}-\sqrt{1-r}\alpha^{*}a_{t}\right){|0\rangle }_{r}{|0\rangle }_{t}\\ ={|\sqrt{r}\alpha \rangle }_{r}{|\sqrt{1-r}\alpha \rangle }_{t} \end{array}$$

The output at the “t” port is coherent state with $\sqrt{1-r}\alpha$ amplitude. The reflectivity of the beam splitter satisfies r+t=1. In this study, the modulation efficiency *M* of the intensity modulator was equal to the transmittance *t* of the beam splitter.

## Appendix C

The MRR-QKD system is divided into the interrogator and MRR terminal, as shown in Figure A4. The interrogator generates an interrogation signal and sends it to the MRR terminal. This interrogation signal is a classical light pulse with fixed circular polarization state (does not carry any information), especially right-hand circular polarization in RM2018. The MRR terminal receives and modulates the interrogation signal and the output signal is in one of states in decoy state BB84 protocol. The modulated signal is retro-reflected back to the interrogator. The interrogator receives and measures the polarization state of the signal.

The interrogation signal is prepared by means of the classical optical pulse with vertical polarization passes through a quarter wave plate oriented at $45^{{}^{\circ}}$. It is necessary to select appropriate signal intensity to ensure that each interrogation pulse contains multiple photons when it reaches the MRR terminal. The interrogation signal is modulated with four pairs of MQWs, which is composed of two stacked MQW. The number of MQWs is eight in total, achieving 15dB extinction ratio of each pair. Each polarization state in BB84 protocol is generated by a set of MQWs and retroreflector in a path respectively, corresponding to H/V(rectilinear basis) and +/− (diagonal basis). The signal in rectilinear basis is generated by the signal transmitted through the BS2; the signal in diagonal basis is generated by the signal reflected by the BS2. It is worth emphasizing that the interrogation signal with right-handed circular polarization is converted into left-handed circular polarization after passing through the half wave plate oriented at 22.5°. The modulated signal with H/V polarization is converted into +/− after passing through the half wave plate.

The transmission loss of each pair of MQWs is controlled independently to realize different states of MQWs. While the transmission loss of the MQWs modulator is set to the lowest (highest), the MQWs is in the “on” (off) state. Polarization modulation is achieved by changing the state of MQWs. For example, if the horizontal polarization(H) is selected, MQWs1 is set in low transmission loss allowing horizontal signal to pass. The other three MQWs are set in high transmission loss which blocks the transmission of signal with unwanted polarization (V/+/−). In this condition, only the horizontal polarization signal can transmit back to interrogator. The decoy state is realized by accurately controlling the transmission loss of MQWs1. Finally, interrogator receives retro-reflected signal and measures its polarization. The measurement process is the same as the traditional QKD based on BB84 [6].

## References

[1] Bennett C.H., Brassard G. (2014). Quantum cryptography: Public key distribution and coin tossing. Theor. Comput. Sci..

[2] Boaron A., Boso G., Rusca D., Vulliez C., Autebert C., Caloz M., Perrenoud M., Gras G., Bussières F., Li M.J. (2018). Secure Quantum Key Distribution over 421 km of Optical Fiber. Phys. Rev. Lett..

[3] Fang X.T., Zeng P., Liu H., Zou M., Wu W., Tang Y.L., Sheng Y.J., Xiang Y., Zhang W., Li H. (2020). Implementation of quantum key distribution surpassing the linear rate-transmittance bound. Nat. Photonics.

[4] Pittaluga M., Minder M., Lucamarini M., Sanzaro M., Woodward R.I., Li M.J., Yuan Z., Shields A.J. (2021). 600-km repeater-like quantum communications with dual-band stabilization. Nat. Photonics.

[5] Nauerth S., Moll F., Rau M., Fuchs C., Horwath J., Frick S., Weinfurter H. (2013). Air-to-ground quantum communication. Nat. Photonics.

[6] Liao S.K., Cai W.Q., Liu W.Y., Zhang L., Li Y., Ren J.G., Yin J., Shen Q., Cao Y., Li Z.P. (2017). Satellite-to-ground quantum key distribution. Nature.

[7] Yin J., Li Y.H., Liao S.K., Yang M., Cao Y., Zhang L., Ren J.G., Cai W.Q., Liu W.Y., Li S.L. (2020). Entanglement-based secure quantum cryptography over 1120 kilometres. Nature.

[8] Liao S.K., Cai W.Q., Handsteiner J., Liu B., Yin J., Zhang L., Rauch D., Fink M., Ren J.G., Liu W.Y. (2018). Satellite-Relayed Intercontinental Quantum Network. Phys. Rev. Lett..

[9] Jacobs B.C., Franson J.D. (1996). Quantum cryptography in free space. Opt. Lett..

[10] Chen Y.A., Zhang Q., Chen T.Y., Cai W.Q., Liao S.K., Zhang J., Chen K., Yin J., Ren J.G., Chen Z. (2021). An integrated space-to-ground quantum communication network over 4,600 kilometres. Nature.

[11] Liao S.K., Lin J., Ren J.G., Liu W.Y., Qiang J., Yin J., Li Y., Shen Q., Zhang L., Liang X.F. (2017). Space-to-Ground Quantum Key Distribution Using a Small-Sized Payload on Tiangong-2 Space Lab. Chin. Phys. Lett..

[12] Bedington R., Arrazola J.M., Ling A. (2017). Progress in satellite quantum key distribution. NPJ Quantum Inf..

[13] Oi D.K., Ling A., Vallone G., Villoresi P., Greenland S., Kerr E., Macdonald M., Weinfurter H., Kuiper H., Charbon E. (2017). CubeSat quantum communications mission. EPJ Quantum Technol..

[14] Takenaka H., Carrasco-Casado A., Fujiwara M., Kitamura M., Sasaki M., Toyoshima M. (2017). Satellite-to-ground quantum-limited communication using a 50-kg-class microsatellite. Nat. Photonics.

[15] Villar A., Lohrmann A., Bai X., Vergoossen T., Bedington R., Perumangatt C., Lim H.Y., Islam T., Reezwana A., Tang Z. (2020). Entanglement demonstration on board a nano-satellite. Optica.

[16] Rabinovich W.S., Mahon R., Ferraro M.S., Goetz P.G., Bashkansky M., Freeman R.E., Reintjes J., Murphy J.L. (2018). Free space quantum key distribution using modulating retro-reflectors. Opt. Express.

[17] Rarity J.G., Tapster P.R., Gorman P.M., Knight P. (2002). Ground to satellite secure key exchange using quantum cryptography. New J. Phys..

[18] Vallone G., Marangon D.G., Canale M., Savorgnan I., Bacco D., Barbieri M., Calimani S., Barbieri C., Laurenti N., Villoresi P. (2015). Adaptive real time selection for quantum key distribution in lossy and turbulent free-space channels. Phys. Rev. A.

[19] Wang X., Liu W., Wu T., Guo C., Zhang Y., Zhao S., Dong C. (2021). Free Space Measurement Device Independent Quantum Key Distribution with Modulating Retro-Reflectors under Correlated Turbulent Channel. Entropy.

[20] Wang X.Y., Wu T.Y., Dong C., Zhao S.H., Sun Y. (2021). Prefixed-threshold real-time selection for correlated turbulent channel model for quantum key distribution with modulating retro-reflectors. Quantum Inf. Process..

[21] Stievater T., Rabinovich W., Goetz P., Mahon R., Binari S. (2004). A Surface-Normal Coupled-Quantum-Well Modulator at 1.55 μm. IEEE Photonics Technol. Lett..

[22] Quintana C., Wang Q., Jakonis D., Piao X., Erry G., Platt D., Thueux Y., Gomez A., Faulkner G., Chun H. (2017). High Speed Electro-Absorption Modulator for Long Range Retroreflective Free Space Optics. IEEE Photonics Technol. Lett..

[23] Huang J., Yin Z., Wang S., Li H., Chen W., Han Z. (2012). Effect of intensity modulator extinction on practical quantum key distribution system. Eur. Phys. J. D.

[24] Ghatak A. (2009). Optics.

[25] Bennett C.H. (1992). Quantum cryptography using any two nonorthogonal states. Phys. Rev. Lett..

[26] Toyoshima M., Takenaka H., Shoji Y., Takayama Y., Koyama Y., Kunimori H. (2009). Polarization measurements through space-to-ground atmospheric propagation paths by using a highly polarized laser source in space. Opt. Express.

[27] Gordon K., Fernandez V., Townsend P., Buller G. (2004). A short wavelength GigaHertz clocked fiber-optic quantum key distribution system. IEEE J. Quantum Electron..

[28] Ma X., Qi B., Zhao Y., Lo H.K. (2005). Practical decoy state for quantum key distribution. Phys. Rev. A.

[29] Gobby C., Yuan Z.L., Shields A.J. (2004). Quantum key distribution over 122 km of standard telecom fiber. Appl. Phys. Lett..

[30] Tang Z.-L., Li M., Wei Z.-J., Lu F., Liao C.-J., Liu S.-H. (2005). The quantum key distribution system based on polarization states produced by phase modulation. Acta Phys. Sin..

[31] Wang J.Y., Yang B., Liao S.K., Zhang L., Shen Q., Hu X.F., Wu J.C., Yang S.J., Jiang H., Tang Y.L. (2013). Direct and full-scale experimental verifications towards ground–satellite quantum key distribution. Nat. Photonics.

[32] Schmitt-Manderbach T., Weier H., Fürst M., Ursin R., Tiefenbacher F., Scheidl T., Perdigues J., Sodnik Z., Kurtsiefer C., Rarity J.G. (2007). Experimental demonstration of free-space decoy-state quantum key distribution over 144 km. Phys. Rev. Lett..

[33] Lydersen L., Skaar J. (2010). Security of Quantum Key Distribution with BIT and Basis Dependent Detector Flaws. Quantum Inf. Comput..

[34] Muller A., Herzog T., Huttner B., Tittel W., Zbinden H., Gisin N. (1997). “Plug and play” systems for quantum cryptography. Appl. Phys. Lett..

[35] Zhao Y., Qi B., Lo H.K. (2008). Quantum key distribution with an unknown and untrusted source. Phys. Rev. A.

[36] Peng X., Xu B., Guo H. (2010). Passive-scheme analysis for solving the untrusted source problem in quantum key distribution. Phys. Rev. A.

[37] Nikulin V.V. (2009). Laser communication system with acousto-optic tracking and modulation: Experimental study. Opt. Eng..

